# An Approach to Ring Resonator Biosensing Assisted by Dielectrophoresis: Design, Simulation and Fabrication

**DOI:** 10.3390/mi11110954

**Published:** 2020-10-22

**Authors:** Anders Henriksson, Laura Kasper, Matthias Jäger, Peter Neubauer, Mario Birkholz

**Affiliations:** 1 Institute of Biotechnology, Technische Universität Berlin, Ackerstrasse 76, 13355 Berlin, Germany; peter.neubauer@tu-berlin.de; 2 Department of High Frequency Technology/Photonics, Technische Universität Berlin, Einsteinufer 25, 10587 Berlin, Germany; laura.kasper@tu-berlin.de (L.K.); matthias.jaeger@tu-berlin.de (M.J.); 3 IHP–Leibniz-Institut für Innovative Mikroelektronik, Im Technologiepark 25, 15236 Frankfurt (Oder), Germany; birkholz@ihp-microelectronics.com

**Keywords:** biosensor, microring resonator, photonic sensor, dielectrophoresis, mass transfer, micro fabrication

## Abstract

The combination of extreme miniaturization with a high sensitivity and the potential to be integrated in an array form on a chip has made silicon-based photonic microring resonators a very attractive research topic. As biosensors are approaching the nanoscale, analyte mass transfer and bonding kinetics have been ascribed as crucial factors that limit their performance. One solution may be a system that applies dielectrophoretic forces, in addition to microfluidics, to overcome the diffusion limits of conventional biosensors. Dielectrophoresis, which involves the migration of polarized dielectric particles in a non-uniform alternating electric field, has previously been successfully applied to achieve a 1000-fold improved detection efficiency in nanopore sensing and may significantly increase the sensitivity in microring resonator biosensing. In the current work, we designed microring resonators with integrated electrodes next to the sensor surface that may be used to explore the effect of dielectrophoresis. The chip design, including two different electrode configurations, electric field gradient simulations, and the fabrication process flow of a dielectrohoresis-enhanced microring resonator-based sensor, is presented in this paper. Finite element method (FEM) simulations calculated for both electrode configurations revealed ∇E^2^ values above 10^17^ V^2^m^−3^ around the sensing areas. This is comparable to electric field gradients previously reported for successful interactions with larger molecules, such as proteins and antibodies.

## 1. Introduction

Photonic biosensing based on silicon-on-insulator (SOI) technology has attracted intense interest in the last two decades. The sensors allow for real time responses, a minimal footprint, a high sensitivity (50–500 nm/RIU, [1,2]), and benefits over many biosensor techniques, such as quartz crystal microbalance, electrochemical methods, surface enhanced Raman spectroscopy (SERS), and surface plasmon resonance spectroscopy (SPR), due to their small sizes and scalable mass production, attributed to their compatibility with well-known standardized semiconductor fabrication processes. In addition, photonic integrated circuit technology (PIC) provides the opportunity for the monolithic integration of electronic and photonic devices, allowing the integration of optical sensors, electrodes, detectors, light-sources, and read-out electronics in a single chip. Furthermore, their implementation as biosensors is facilitated by a plethora of well-established and newly developed chemical functionalization protocols that may be applied to immobilize receptor molecules on silicon surfaces [3,4,5].

**Figure 1:**
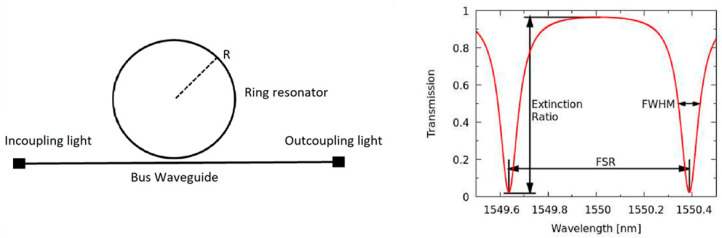
Schematic drawing of the principle of ring resonators and their generated transmission spectrum [8].

SOI-based photonic sensors are generally realized as interferometers (often in the form of a Mach–Zehnder interferometer) or resonators (often in the form of microring resonators), in which the latter benefit from higher sensitivities and small sizes, making them more suitable for integrated sensing platforms [6]. As first proposed in the mid-90s, ring resonator technology is based on the looped propagation of light in the form of whispering gallery modes, creating a resonance at frequencies fulfilling the resonance conditions. The light traveling through the waveguides remains within the waveguides due to total internal reflection and generates an evanescent field near the waveguide surface, whose interaction with the environment enables the sensing of analytes. In the simplest devices, a looped waveguide (ring resonator) with a loop diameter in the range of a few 10 to a few 100 µm is placed adjacent to a linear waveguide (bus). As light propagates through the straight waveguide, from the incoupling to the outcoupling fiber, wavelengths (resonance wavelengths) with a harmonic relation to the ring’s circumference of 2π*Rn* = *m*λ_r_ (m being the mode number, R the ring radius, λ_r_ the resonance wavelengths, and n the effective refractive index) are trapped in the ring (Figure 1) [7].

Organic molecules, such as proteins immobilized on the ring, cause *n* around the resonator to change—represented as Δ*n*—so longer wavelengths of light are trapped. Transmission spectra with resonance wavelengths λ_r_ and the sensing-induced resonance shift are subsequently monitored by photodetectors [9].

Ring resonators with different configurations and geometries have been developed to improve the sensing ability, reduce the impact of production tolerances, and improve the detection limit [9]. These include the coupling of two linear waveguides to form a single microring, a two-cascade ring resonator (Vernier-cascade silicon photonic label-free biosensor) [10], and a coupled-resonator optical waveguide (CROW) that is an array of optical cavities, where adjacent cavities are coupled to each other and thereby amplify the response [11].

Typically, a microring resonator allows protein detection in the picomolar (pM) range on a 10–30-min timescale [9,12,13]. This is comparable to other optical nano-scaled sensors, such as SPR and SERS [14,15], and during the last decade, a large variety of bioanalytical applications have been reported, including protein diagnostic assays [16], protein interaction studies [17], and the detection of viruses [18] and bacteriophages [19,20].

Plasmonic enhancements have further reduced the limit of detection to even lower levels, allowing single protein detection at concentrations in the femtomolar (fM) range. Furthermore, even single macromolecular detection in the attomolar (aM) range was reported for a microtoroid whispering gallery mode geometry by an approach called frequency locking [21].

A limiting factor for the sensitivity of a ring resonator, as with all receptor-based biosensors, is the diffusion of analytes from the bulk solution to the sensor surface. This was nicely illustrated by Squirres et al. [22], who only calculated a single binding event every 3 days in a sensor, modeled as a nanowire with dimensions in the nanometer range and a target protein solution with a concentration of 10 fM. In order to increase the transfer rate and thus the detection sensitivity, techniques that facilitate the movement of bioparticles to the sensing electrode are required. Microfluidic systems have turned out to be an effective alternative for delivering the sample as they allow the convenient handling of volumes in the µL range.

Among other methods employed to manipulate the mass transfer of biomolecules, such as mechanical manipulation [23], inertial lift forces [24], hydrodynamic techniques [25], acoustic techniques, optical tweezers [26], magnetic manipulation [27], electrophoresis, and dielectrophoretic forces, the latter have many advantages, due to their versatile strong interactions with neutral particles and straightforward integration into microfluidic systems [28].

Dielectrophoresis (DEP) is the migration of polarized dielectric particles in a non-uniform electric field and has attracted intense interest in biotechnology regarding the separation and concentration of viruses [29], bacteria [30], micro algae [31], DNA [32], and protein [33], as well as its compatibility with micro fluidic systems and lab-on-a-chip (LOC) devices [33,34].

An induced dipole interacts with the electrical field gradient ∇E and depending on the relative polarizability of the particle and the suspending medium, an attractive or repulsive force may be induced on the particle, which will subsequently move towards (positive DEP) or away from (negative DEP) the strongest generated electric field. The dielectrophoretic force is correlated with the electrical conductivity σ and dielectric constant ε of the particle, which represent various structural, morphological, and chemical characteristics, hence enabling a highly selective manipulation.

Furthermore, in contrast to electrophoresis, where only charged particles can be manipulated, dielectrophoresis may also be implemented for interactions with uncharged analytes [35].

While dielectrophoresis has mainly been applied for the manipulation of larger particles, there are also several investigations dealing with molecular dielectrophoresis. For example, several interactions with proteins and oligonucleotides have been reported, as pioneered by Wahizu [36] et al. and Hölzel et al. [37].

Since the induced force is proportional to the cubic radius of the particle, i.e., its volume, molecular dielectrophoresis is challenging. To achieve the same dielectrophoretic effect as for larger particles, this may be compensated for by applying higher voltages, placing the electrodes closer together, and optimizing the geometry to achieve high electric fields and gradients [35,38].

As nano-technological fabrication methods have improved, several successful electrode configurations have been developed for interactions with molecules. These include individual electrode pairs, interdigitated electrodes, quadrupole electrodes, and 3D electrode arrays [39].

It has also been shown that DEP and bio-sensing may be combined with great success. Li et al. [40] reported a capacitive immunosensor whose performance was substantially increased after focusing the analyte on the sensor surface and Freedman et al. [41] recently reported a 1000-fold improved detection efficiency in nanopore sensing using DEP trapping. Similar effects combined with optical label-free sensing methods, such as a typical microring resonator, would allow for protein detection in the attomolar range.

This triggered us to explore microring resonance biosensing assisted by dielectrophoretic forces, as such a combination of these successful approaches, to the best of our knowledge, has not yet been explored. In the following work, we present the design, simulation, and fabrication process of a ring resonator with a biosensing functionality assisted by positive dielectrophoresis. We chose to explore two electrode configurations for this purpose: One with a coplanar single electrode pair enclosing the sensor area and one 3D geometry, with one electrode in the immediate proximity to the sensing surface on the sensor chip and the second electrode placed on top of a microfluidic channel.

## 2. Materials and Methods

### 2.1. Fabrication

SOI consists of a bulk silicon support wafer and a buried oxide layer as an electric insulator (SiO_2_) that separates the bulk silicon substrate from a second silicon layer, often called the device layer. In a typical application, sensing elements, waveguides, and possible integrated circuit (IC) devices are built on the device layer, with the buried oxide functioning as an effective etch-stop during wafer processing.

In an SOI waveguide, light with a wavelength of about 1550 nm is guided in silicon as a high index medium (n = 3.476) called the core (Figure 1), separated from the silicon substrate by the buried oxide as a low index medium (n = 1.444). Above and on the side of the waveguide is an upper cladding layer of a low index medium (often air or SiO_2_), allowing the light to propagate via total internal reflection.

**Figure 2:**
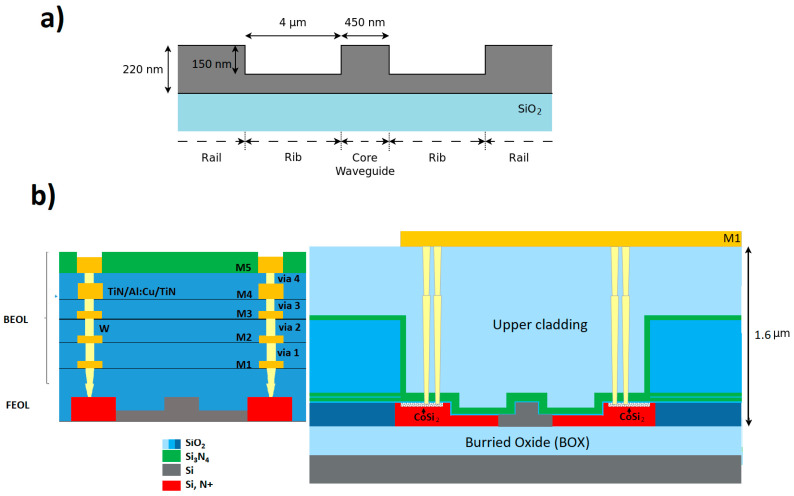
(**a**) Cross-section of the nano rib waveguide structure used in this work. (**b**) Schematic figure of the general IHP photonic BICMOS architecture. In this work, the wafers were only processed up to the first metallization layer.

In this work, the photonic chip was fabricated in a photonic integrated circuit (PIC) using a 200 mm SOI wafer with a 220 nm thick Si device layer on top of a 2 µm buried oxide. The back-end of line (BEOL) allowed individual components comprising three thin and two thick metal layers, named m1 to m5 from bottom to top, to be interconnected. However, the wafers were only processed up to the first metallization layer (Figure 2).

Metal layers were essentially made of Al:Cu exhibiting thicknesses from 0.5 to 2 μm and sheet resistances of approx. 10 m·Ω·sq ^−1^. The Al:Cu alloy was confined between 50 nm thin TiN layers, in order to suppress the oxidation of Al and to avoid the out-diffusion of Cu.

The waveguides prepared for this work were standard rib waveguides with a core width of 450 nm, a core height of 220 nm, a 70 nm rib height, and a rail height of 220 nm. For the manufacturing, standard SOI processing, including photolithograpy and inductively coupled plasma etching, was used.

Doped waveguides were prepared via ion implantation. Furthermore, silicidation of the rail with cobalt silicide (CoSi_2_) allowed the waveguide to be connected to the metal layer through a tungsten vertical interconnect access (VIA).

The cladding over the waveguides was a stack of 10 nm silicon dioxide directly on top of the SOI silicon layer, a 100 nm silicon nitride layer, and finally silicon dioxide with a thickness of 1.6 µm. The resonators were further exposed in the sensing areas by removing the cladding stack with reactive ion etching (RIE) over a resist mask.

### 2.2. Simulation by the Finite Element Method (FEM)

The induced dipole interacts with the electrical field gradient ∇E, resulting in the dielectrophoretic force ***F****_DEP_*, which depends on the polarizability of the particle (index *p*) within the medium (*m*), where the latter can be expressed by a function of their complex dielectric constants. Accordingly, ***F****_DEP_* exerted upon a spherical particle in an *AC* field can be given by

F_DEP_ = 2πε_m_r^3^Re(f_cm_) ∇E^2^, (1)

where r is the particle radius and real part of the complex Clausius–Mossotti factor Re(f_cm_) describing the dielectric properties of the particle and the medium and ∇E^2^ is the generated gradient of the squared electric field.

From Equation (1), it may be concluded that, apart from changing the size and dielectric properties of the particles, the dielectrophoretic force may be influenced by the generated ∇E^2^. The ∇E^2^ may be tuned by changing the electrode geometry and applied voltage and may be calculated by using FEM to solve Maxwell’s equations.

In order to enable an estimation of the performance of the device, simulations with FEM of ∇E^2^ in the y direction were conducted using the electrostatic module in Comsol Multiphysics by employing the expression “log(sqrt(d(ec.normE^2,y)^2))/log(10)” and the material properties given in Table 1.

## 3. Results

### 3.1. Electrode Design and Simulation of the ∇E^2^ Distribution

#### 3.1.1. 3D Electrode Geometry

The electrodes and ring resonator layout were designed to be compatible with the IHP PIC technology using a commercially available 200 mm SOI wafer with 2 µm thick SiO_2_ and a 220 nm thick top silicon layer.

Among the different types of nano waveguides that can be produced, we chose the nano rib waveguide, as it has previously been prepared for biosensor purposes with good results [42].

Moreover, the IHP photonic BiCMOS technology further allows for ion implantations and the preparation of doped rib waveguides (n^+^, p^+^) that can be connected via the metal 1 layer, as has been previously shown for the preparation of optical pn phase shifters [43,44].

This approach was applied in this work, in order to realize a DEP electrode adjacent to the sensing segment of the ring resonator.

**Figure 3:**
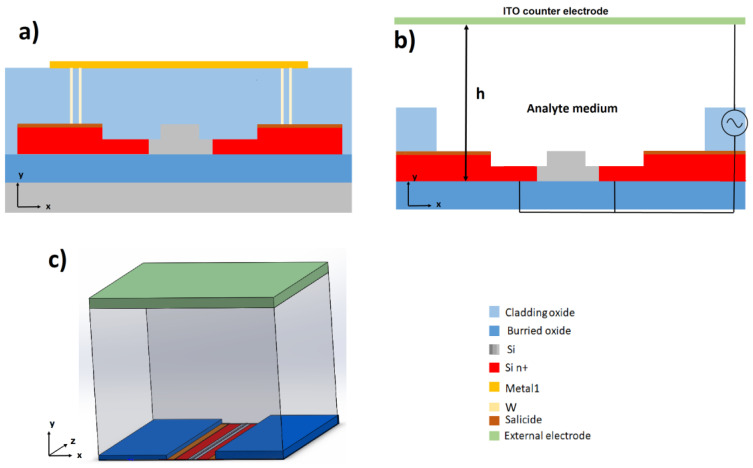
(**a**) Contacting the silicon waveguide: Cross-section of the silicon waveguide (xy plane) used for the 3D electrode geometry. The red areas represent highly-doped silicon that is contacted via a channel from the metal 1 layers. Most of the waveguide is covered by an SiO_2_ layer to protect it from the outside and prevent losses. (**b**) In the sensing areas, the SiO_2_ is etched off, to allow exposure of the waveguide to the analyte medium. The doped area on the waveguide functions as a dielectrophoresis (DEP) electrode, whereas the counter electrode is placed on top of a microfluidic channel with the distance h in the y-direction to the waveguide. (**c**) 3D view of the sensor region.

As shown in Figure 3a, the waveguides were covered by a cladding SiO_2_ layer to prevent optical losses. The red areas represent highly n-doped silicon with a resistivity of approximately 5 mΩcm that can be contacted via the metal 1 layer. The core waveguide is kept lightly p-doped with a resistivity of 10 Ωcm, which is required to preserve the optical properties.

In Figure 3b, which illustrates the sensing area, the protecting SiO_2_ is etched off to expose the waveguide to the medium and the analytes to be detected. The counter electrode is made of a transparent conductive oxide (TCO), such as indium thin oxide (ITO), and is placed on top of a microfluidic channel, 50–200 µm above the waveguide. As this electrode is several dimensions larger than the waveguide, an inhomogeneous electric field with its maximum on the waveguide surface may be generated. Compared to other common electrode geometries that generate a strong tangential field vector E, the electric field vector in the 3D geometry is primarily oriented orthogonal to the surface. As the tangential field may lead to electro-osmotic fluid flow, which interferes with the application of the DEP effect, this geometry has been proposed to be suitable for protein dielectrophoresis [37].

**Figure 4:**
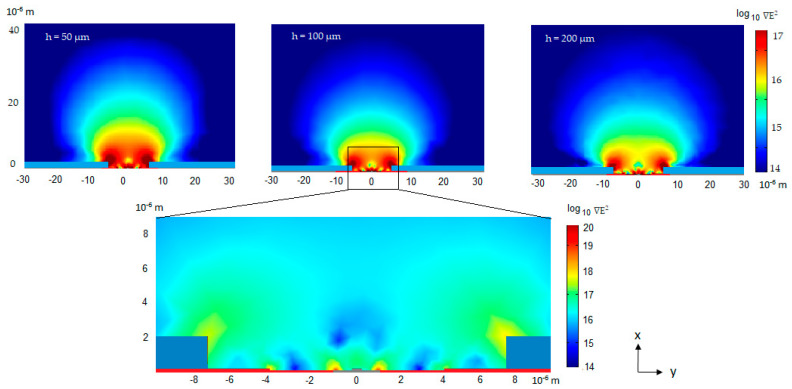
Finite element method (FEM) simulations, as described in Section 2.2, of the ∇E^2^ distribution in the 3D geometry with a 10 V peak to peak potential applied, given in V^2^m^−3^. The figure shows the cross-section (xy plane) at different heights h in relation to the counter electrode. The waveguide is placed in center at the bottom, extending in the z-direction. The analytes are expected to be transported from the bulk solution to the area with the strongest ∇E^2^, next to the core waveguide.

In Figure 4, FEM simulations of the ∇E^2^ distribution on the xy plane, corresponding to geometry of Figure 3b, are shown. The peak to peak potential is 10 V and ∇E^2^ in the order of 10^14^ V^2^m^−3^ can be observed approximately 50 µm above the sensor. The ∇E^2^ increases with a decreasing distance to the waveguide, reaching a maximum above 10^20^ V^2^·m^−3^ close to the core waveguide surface. As the analytes are expected to interact with the applied field and move to the highest ∇E^2^, they are expected to be transported from the bulk solution to the sensor surface, thus increasing the sensing ability. Changing the distance (h) of the counter electrode from 50 to 200 um slightly reduces the generated electric field strength. However, the change is not dramatic, suggesting that the sensor is feasible, even with the counter electrode placed at greater distances. Being able to place the electrode further away from the sensor surface may ease the packaging and the integration into a microfluidics system.

#### 3.1.2. Coplanar Electrode Pair Configuration

**Figure 5:**
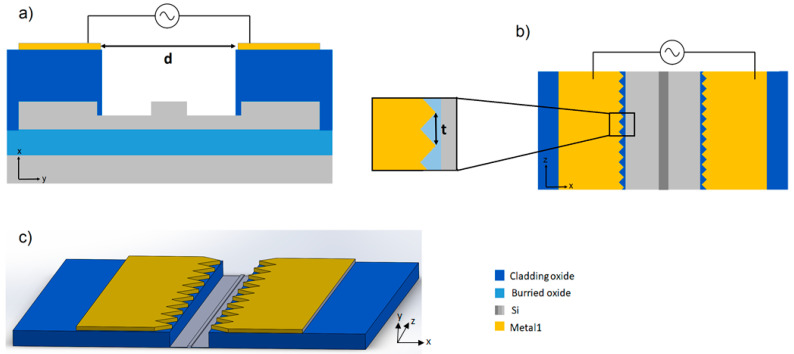
(**a**) Cross-section of the sensing region with the coplanar electrode pair geometry. (**b**) Top view of the coplanar electrode pair geometry. (**c**) 3D view of the sensing region.

A cross-section and a top-view of the electrode pair layout are illustrated in Figure 5a,b. Two electrodes are realized as part of the metal layer 1 (M1), 1.6 um above the silicon waveguide, as shown in Figure 5a. The electrodes enclose the exposed waveguide that is the sensor surface. As the highest fields are generated at sharp edges, a thorny-shaped electrode was designed, with the highest electric fields generated on the thorn-edges and spreading over the electrode gap. Due to the fabrication process, the angle α at the thorn was restricted to a minimum of 45°.

**Figure 6:**
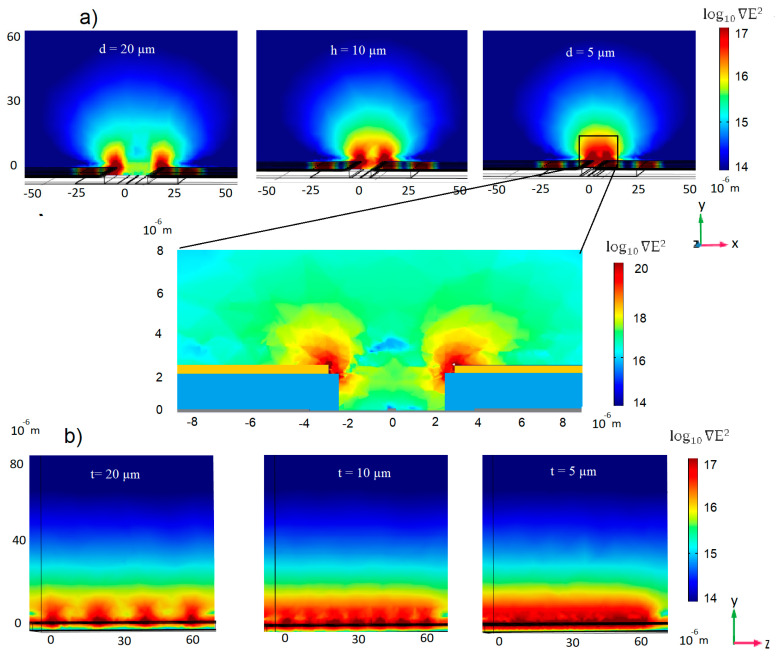
(**a**) Cross-section (xy plane) of the coplanar electrode configuration with different distances (d) between the electrodes. The waveguide is in the center at the bottom, extending in the z-direction (**b**) Cross-section (yz plane) at the center of the waveguide with the coplanar electrode configuration. Simulated ∇E^2^ distribution at different thorn-to-thorn distances (t).

FEM simulations were conducted for the optimization of the electrode gap (distance between the electrode pair), so that a sufficient electric field was generated above the sensor surfaces. As shown from the FEM simulations of the xy plane (Figure 6a), a large distance d between the electrodes only generates electric fields above 10^17^ V^2^·m^−3^ at the electrode edges. Bringing the electrodes closer together allows a high field gradient in the whole area between the electrodes to be generated at reasonable applied voltages; here, at a 10 V peak to peak potential. The purpose of this design is to transport analytes from the bulk solution closer to the sensor surface, thus locally increasing the analyte concentration. A similar electrode configuration has previously been reported to lower the detection time [45] from 60 to 1 min and increase the limit of detection of nanowire field effect transistors by a factor of 10^4^, reaching the aM range [46]. The mechanism was explained as a combination of AC electro-osmosis and streaming DEP, enabling a DEP interaction with proteins that cannot be immobilized against the electro-osmotic conveyance. Consequently, the proteins will concentrate and rarefy into flowing filamentary streams.

In order to achieve a strong ∇E^2^ over the whole sensor surface, the distance t between the thorn edges should be small, as shown in Figure 6b. Here, the ∇E^2^ is simulated on the yz plane along the center of the waveguide. At larger distances, an inhomogeneous field is observed. At shorter distances, on the other hand, the generated ∇E^2^ above 10^17^ V^2^·m^−3^ is homogenously distributed over the sensor surface.

From these simulations, we decided to choose an electrode gap and a thorn-to-thorn distance of 5 µm.

### 3.2. Ring Resonator Design

**Figure 7:**
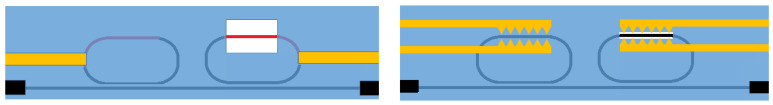
Ring resonator layout. Each sensor consists of two ring resonators (dark blue) coupled to a common bus waveguide (dark blue) using directional couplers. One ring is completely covered with SiO2 (light blue) and acts as a reference to control for ambient temperature. The two variants differ in terms of using doped waveguides (left) and coplanar electrodes (right) for dielectrophoresis.

Each sensor consists of two ring resonators coupled to a common bus waveguide using directional couplers (Figure 7). One ring is completely covered by cladding SiO_2_ and acts as a reference to control for ambient temperature variations. For the other ring, the passivating SiO_2_ layer is locally etched off, exposing part of the waveguide to the analyte solution, and used as a sensor.

Two grating couplers at the ends of the bus waveguide allow for coupling to fiber optics. A racetrack-like ring geometry with two 90 µm straight segments parallel to the bus function as a directional coupler and sensing area, respectively; a 20 µm straight segment vertical to the bus allows for electrical contact with the waveguide; and four 90° bends (radius of 55 µm) connect the straight segments. A straight sensor area was chosen, as it allows for a straightforward way of placing the coplanar electrode pair.

Furthermore, this geometry allows for both electric contact with the rib and a spatial distance between the placed electrodes and the directional coupler, thus avoiding interference by the AC field in the coupling region. This helps in ensuring a straight forward evaluation of spectra measured while the electrodes are in use, as any refractive index change induced by the electrodes (e.g., by temperature changes) will only influence the wavelength at which resonance occurs and not its extinction ratio. That would be the case if the effective index in the coupling region changed. Limited changes to the extinction ratio are still possible due to changes in absorption because of the presence of the sample and concentration of analyte in the exposed area and artefacts caused by integration occurring during the acquisition of the optical transmission spectrum.

Exposing only part of the sensing ring serves several purposes. Firstly, it simplifies the electrical contact, as it allows for the circuit on the M1 layer to reach the center of the ring. Secondly, it keeps the sample from filling the volume around the directional coupler, where changes to the refractive index would influence the coupling factor and thereby also the extinction ratio. Such changes would make it hard to ensure that the system performs comparably in both the dry state and wet state [47]. The size of the opening is chosen such that it only contains the straight part of the wave-guide, in order to optimize the design for producing a simple geometry and a more explainable system, without bent electrodes. The system sensitivity can be increased linearly with the portion of the ring exposed and accessed by electrodes.

As the operation of the DEP influences the resonance wavelength via the thermo optic effect, measured values are only compared for the same state of electrodes. This allows for both a comparison of measurement values before and after the binding of the analyte, as well as monitoring of the binding process itself while the DEP voltage is applied. Depending on the oscillation frequency of the DEP voltage and the strength of the thermoelectric effect, integration times during the acquisition can lead to an apparent widening of the resonance dips. In this case, a lock-in amplifier locked to twice the modulation frequency can be used to more precisely measure the resonance wavelength [47].

The radius of the circular segment was set to 55 µm, as for the given circumference, a larger radius would have decreased the coupling and sensing areas. Too small bend radii, on the other hand, cause optical losses [8].

A waveguide geometry consisting of rib waveguides of a 450 nm core width, 220 nm core height, 70 nm rib height, and 4 µm rib width was chosen (Figure 5b). Similar structures have previously been shown to possess optical properties suitable for biosensing, with a bulk sensitivity of 90 nm/refractive index unit (RIU) (without cladding) and 28 nm/RIU (with cladding and an opening exposing the waveguide) and a quality-factor (defined as r/FWHM) of around 1.3 × 10^5^ [48]. A free spectral range (FSR) of approximately 1 nm was chosen, in accordance with the same study. This ensures that multiple resonances are visible within typical scanning ranges of tunable laser sources (TLS), whilst at the same time ensuring that the FSR is large enough that the binding events of interest do not change the resonance frequency by more than one FSR, guaranteeing that the shift measured using measurements before and after the binding event is unambiguous. If only research-grade TLS are used, a larger FSR allowing for a larger unambiguous measurement range is possible, but that would also require a smaller ring circumference, limiting the possibility to separate the bent sections, the coupling section, the interaction section, and the electrical contact section of a ring.

The width of the doping areas was set to 6.5 µm at a 775 nm distance to the core waveguide. Doping was applied along approximately half of the ring resonator, starting at the electrical contacts and ending after the sensing region, in order to prevent interference at the directional couplers.

### 3.3. Fabrication and Optical Characteristics

**Figure 8:**
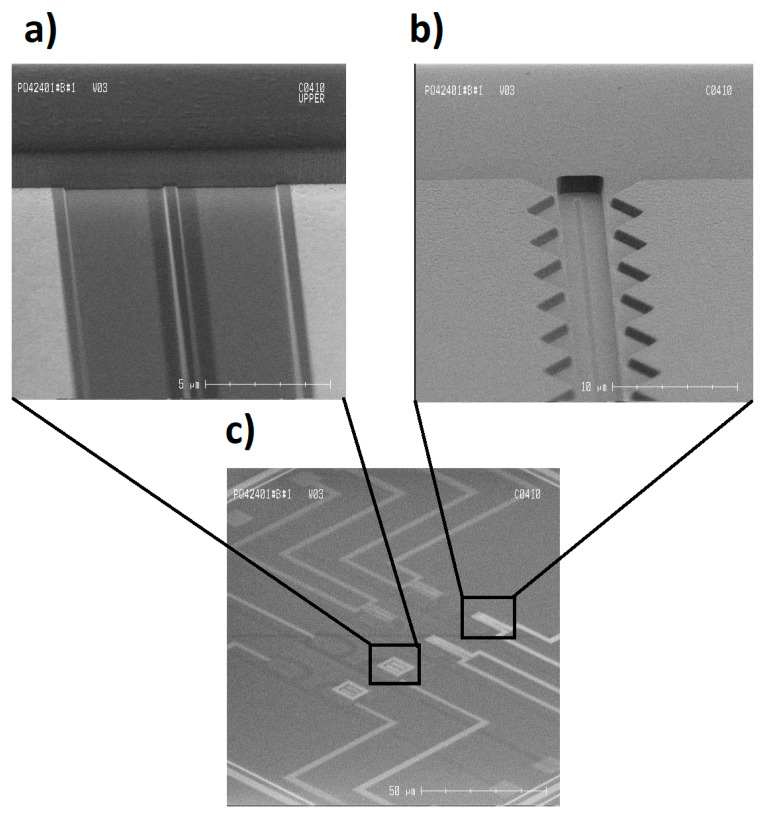
SEM image of the fabricated sensors. (**a**) The sensor region in the doped waveguide, corresponding to Figure 3c. The core waveguide can be observed at the center as a bright line next to a thin dark gray area representing the undoped rib. The brighter gray area is attributed to the doped rib, while the whiter region is attributed to the salcide segment. (**b**) The coplanar electrodes: A 4 × 90 µm large window that is etched with reactive ion etching (RIE) that exposes the core waveguide can be observed at the center of the opening. (**c**) An overview of the chip layout, showing the ring resonators, sensing areas, and electrode structures.

The silicon-based photonic microring resonators were successfully constructed in a photonic integrated circuit (PIC) using a 200 mm SOI wafer with a 220 nm thick Si device layer on top of a 2 µm buried oxide. In Figure 8a,b, SEM images of the sensing regions of the fabricated sensors are shown, whereas an overview of the layout can be seen in Figure 8c.

**Figure 9:**
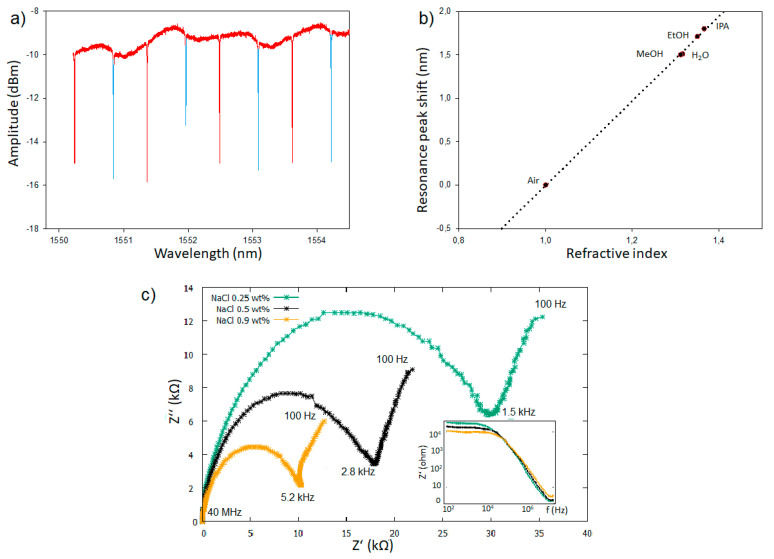
(**a**) A typical experimental transmission spectrum of the ring resonators. The red peaks are attributed to the reference ring and the blue to the sensor ring. (**b**) The resonance peak shift of the ring resonators exposed to MeOH (n = 1.3118), [51] H_2_O (n = 1.3164), EtOH (n = 1.3503), and isopropanol (n = 1.3661) normalized to the peak position in air (n = 1.003). [52] (**c**) Nyquist and Bode plot representing the impedance measurement of NaCl aqueous solutions, as performed with the coplanar electrode configuration.

The experimental transmission spectra of three typical devices (Figure 9a) were taken for characterization. Each sensor generates two resonance peaks (one attributed to the reference ring and one to the sensing ring) that are harmonically repeated at frequencies that fulfil the resonance conditions [1,49].

The free spectral range (FSR), as the spectral distance between two adjacent resonances (different resonance mode) of the same ring resonator, was determined to be 1.12–1.13 nm and the Q-factor was determined to be approximately 1–1.5 × 10^5^. The extinction ratio of the waveguides was determined to be 3–7 dB for both the reference ring and the exposed ring.

The variations in the extinction ratio may be caused by small reflections, resulting in coupling to the back-propagating mode of the ring resonator.

The sensitivity of a completely exposed ring resonator is given by Equation (2):

(2) $$S_{vol}=\frac{\delta \lambda }{\delta n_{vol}}=\frac{\lambda }{n_{g}(\lambda ,\;n_{vol})}\frac{\partial n_{eff}(\lambda ,\;n_{vol})}{\partial n_{vol}},$$

where the ambient volume index sensitivity *S_vol_* is defined as the change in the spectral position of a resonance peak per change of the ambient volume refractive index n_vol_, n_eff_ is the effective index of the light propagating in the waveguide, and n_g_ is the effective group index for the light propagating in the waveguide.

With the simulation-based values taken from [8] of n_g_ = 3.7591 and ∂*n_eff_*//∂*n_vol_* = 0.0720 at a wavelength of λ = 1550 nm and a nanorib waveguide of the same dimensions as the design values used in this work, this results in an expected value of 29.4 nm/RIU for a ring resonator that is completely exposed to the sample. In this case, only a 90 nm section of the whole circumference of 550 nm is exposed, which translates to a sensitivity of 4.82 nm/RIU.

The resonance peak positions are in a first order approximation of Equation (2) for small changes of the ambient refractive index and linearly correlated to the refractive index of the environment with a sensitivity of 4.9 ± 0.03 nm/RIU for the ring resonator with the top bottom electrode configuration, as shown in Figure 9b, and 2.7 ± 0.1 nm/RIU for the coplanar electrode configuration (see supporting information Figures S1–S3). The measured sensitivity is larger than the expected theoretical value by more than the statistical measurement error of the sensitivity measurement. This difference is attributed to production tolerances in the shape of the waveguide, such as the waveguide width or the etch depth. The reason for the reduced sensitivity of the ring resonator with the coplanar electrode configuration is likely that the sides of the core waveguide are still partly covered by silicon nitride as the narrow sensing window makes the etching process conducted to expose the waveguide less efficient.

It is acknowledged that the yet unoptimized sensitivity reduces the measurement capabilities of the realized system. The exact characteristics have been chosen as they are used to reduce the complexity and ensure reproducibility of the system. In the further development of a measurement system based on this work, the sensitivity can be increased to 29.4 nm/RIU by using the full circumference of the ring. For the coplanar electrode configuration, it could be possible to further increase the sensitivity through a switch to nanowire waveguides. Nanowire waveguides provide a higher field penetration in the cladding region compared to rib waveguides, thus enabling a stronger interaction with the analyte and a higher sensitivity. According to simulation-based values [8], 450 nm wide and 220 nm high nanowire waveguides would result in a new sensitivity of 69.2 nm/RIU.

Finally, the functionality of the electrodes was verified by measuring the impedance between 100 and 40 MHz of NaCl dissolved in milli-Q water. In accordance with the literature [50], the Bode plot of the impedance measurements Z’(*f*) showed a large plateau between 100 and 10^4^ Hz, with the value of the plateau increasing with a decreasing salt concentration. Furthermore, the Nyquist plot displayed a characteristic shape of Randle equivalent circle and the expected trend as the electrolyte charge transfer resistance (represented by the semi-circle part of the plot) increased linearly with the increased salt concentration.

## 4. Discussion

In this work, ring resonators with integrated electrodes in close proximity to the waveguide aimed at dielectrophoretic-assisted biosensing were successfully designed and fabricated using photonic integrated circuit (PIC) technology. The chip design, with two different electrode configurations and electric field gradient simulations, was presented. In the first design, the rib and the rail next to the core waveguide were used as an electrode, whereas the second electrode was placed on top of a microfluidic channel. In the second design, an electrode pair was placed on the metal 1 layer enclosing the sensor surface. FEM simulations calculated for both electrode configurations showed a ∇E^2^ in the 10^16^–10^20^ V^2^m^−3^ range around the sensing MRR areas. This is comparable to values previously reported for successful interactions with larger molecules, such as proteins and antibodies [37,39].

The strong simulated ∇E^2^ suggests that the electrodes can be applied to the ring resonator surface to directly target biomolecules, thus bypassing limitations in diffusion kinetics and improving the sensitivity in biosensor applications.

While the purpose of both electrode geometries is to focus analyte molecules on the sensing MRR surface to improve receptor-based sensing, the ∇E^2^ generated also suggests that the dielectrophoretic force may assist in surface functionalization. The application of electrical forces for permanently bonding receptor molecules to the sensor surface and thereby simplifying biofunctionalization has already been shown for other mesoscopic surface structures [53]. In the case of dielectrophoresis, the induced surface immobilization would be applicable in a 3D electrode geometry, where the highest electric fields are generated very close to the sensing region.

As the highest field strengths in the coplanar electrode configuration are not generated on the microring directly, but on the metal 1 layer, DEP-assisted surface immobilization is precluded. However, this configuration may be applied to increase the concentration of analytes through streaming dielectrophoresis. The coplanar electrode geometry further benefits from an easier integration in microfluidics. In this work, a rib waveguide was chosen for the realization of ring resonators. This geometry offers advantages, such as low losses, less dependence on the production tolerance, and compatibility with both electrode configurations. However, it suffers from a lower bulk sensitivity compared to other waveguide geometries, such as the nanowire waveguide or the slot waveguide, as the evanescent field is not penetrating as far into the cladding layer [8,9]. Therefore, a further increase in sensitivity may be achieved by optimizing the waveguide structure. While the coplanar electrode geometry is compatible with most available waveguides, the top-down arrangement requires several new solutions to realize the electric contacts. Variations of the waveguides that could be straight-forwardly implemented to increase the sensitivity would include both nano wire or slot structures for the coplanar electrode geometry and a one-sided nanorib structure for the top-bottom arrangement.

Dielectrophoresis has been frequently studied for the manipulation of oligonucleotides [54]. Furthermore, at least 22 globular proteins with a radius in the range 2–7 nm have been investigated [55]. Therefore, the realized sensor may be beneficial as both a genosensor and immunosensor for applications that require an improved sensitivity and kinetics.

While the sensors were designed to assist with molecular biosensing, they may also be beneficial for the detection of whole cells and pathogens. There are only a few examples of the MRR analysis of whole cells, such as *E. coli* and salmonella, and they have shown a low sensitivity that can be partly explained by non-specific bindings and a small number of analytes [56,57]. Dielectrophoresis may be a solution to this problem, due to its strong specific interaction with cells that may enable the target cells to be trapped on the sensor surface. The attractiveness of such an approach could be the objective for directly and quantitatively detecting cells and pathogens with minimal sample preparation. Furthermore, dielectrophoresis allows discrimination between living and dead cells, which is of great importance for various applications, e.g., the detection of legionellae in drinking water [58].

The design and fabrication work presented represents, to the best of our knowledge, the first attempt to combine the two techniques of SOI-based photonic sensors and dielectrophoresis. Therefore, this approach may lead to new strategies for detecting biological events, as well as expand the applications of photonic sensors and reduce the detection limits.

## Figures and Tables

**Table 1:** Material properties used in finite element simulations.

Material	Relative Permittivity	Electric Conductivity (S/m)
SiO_2_	3.74	10^−14^
Medium (PBS Puffer)	80	1
Si	11.7	10
n^+^-Si	11.7	2 × 10^4^
Metal 1	1	10^5^

## Supplementary Materials

The following are available online at https://www.mdpi.com/2072-666X/11/11/954/s1, Figure S1: Typical transmission spectrum of ringresonators with coplanar electrode configuration; Figure S2: Typical transmission spectrum of ringresonator with top-bottom electrode configuration; Figure S3: The resonance peak shift of the ring resonators with coplanar electrodes exposed to MeOH (n = 1.3118), H_2_O (n = 1.3164), EtOH (n = 1.3503), and isopropanol (n = 1.3661) normalized to the peak position in air.

## References

[1] Sarkaleh A.K., Lahijani B.V., Saberkari H., Esmaeeli A. (2017). Optical Ring Resonators: A Platform for Biological Sensing Applications. J. Med. Signals Sens..

[2] Birkholz M., Mai A., Wenger C., Meliani C., Scholz R. (2015). Technology modules from micro- and nano-electronics for the life sciences. Wiley Interdiscip. Rev. Nanomed. Nanobiotechnol..

[3] Lummerstorfer T., Hoffmann H. (2004). Click Chemistry on Surfaces: 1,3-Dipolar Cycloaddition Reactions of Azide-Terminated Monolayers on Silica. J. Phys. Chem. B.

[4] Henriksson A., Nishiori D., Maeda H., Miyachi M., Yamanoi Y., Nishihara H. (2018). Attachment chemistry of aromatic compounds on a Silicon(100) surface. Surf. Sci..

[5] Henriksson A., Hoffmann H. (2018). Click Coupling Reactions on Flat and Nanostructured Hydrogen-Passivated Silicon Surfaces. Phys. Status Solidi.

[6] Wang J., Sanchez M.M., Yin Y., Herzer R., Ma L., Schmidt O.G. (2020). Silicon-Based Integrated Label-Free Optofluidic Biosensors: Latest Advances and Roadmap. Adv. Mater. Technol..

[7] Bogaerts W., De Heyn P., Van Vaerenbergh T., De Vos K., Kumar Selvaraja S., Claes T., Dumon P., Bienstman P., Van Thourhout D., Baets R. (2012). Silicon microring resonators. Laser Photon. Rev..

[8] Jäger M. (2016). Spatially Resolved Refractive Index Detection Based on SOI Ring Resonators. Ph.D. Thesis.

[9] Steglich P., Hülsemann M., Dietzel B., Mai A. (2019). Optical Biosensors Based on Silicon-On-Insulator Ring Resonators: A Review. Molecules.

[10] Claes T., Bogaerts W., Bienstman P. (2011). Vernier-cascade silicon photonic label-free biosensor with very large sensitivity and low-cost interrogation. Biosensing Nanomed. IV.

[11] Yariv A., Xu Y., Lee R.K., Scherer A. (1999). Coupled-resonator optical waveguide: A proposal and analysis. Opt. Lett..

[12] Luchansky M.S., Bailey R.C. (2010). Silicon Photonic Microring Resonators for Quantitative Cytokine Detection and T-Cell Secretion Analysis. Anal. Chem..

[13] Su J. (2017). Label-Free Biological and Chemical Sensing Using Whispering Gallery Mode Optical Resonators: Past, Present, and Future. Sensors.

[14] Huertas C.S., Calvo-Lozano O., Mitchell A., Lechuga L.M. (2019). Advanced Evanescent-Wave Optical Biosensors for the Detection of Nucleic Acids: An Analytic Perspective. Front. Chem..

[15] Nguyen A.H., Peters E.A., Schultz Z.D. (2017). Bioanalytical applications of surface-enhanced Raman spectroscopy: De novo molecular identification. Rev. Anal. Chem..

[16] Kindt J.T., Bailey R.C. (2013). Biomolecular analysis with microring resonators: Applications in multiplexed diagnostics and interaction screening. Curr. Opin. Chem. Biol..

[17] Ksendzov A., Lin Y. (2005). Integrated optics ring-resonator sensors for protein detection. Opt. Lett..

[18] Zhu H., White I.M., Suter J.D., Zourob M., Fan X. (2008). Opto-fluidic micro-ring resonator for sensitive label-free viral detection. Analyst.

[19] Zhu H., White I.M., Suter J., Fan X. (2008). Phage-based label-free biomolecule detection in an opto-fluidic ring resonator. Biosens. Bioelectron..

[20] Bahadoran M., Noorden A.F.A., Mohajer F.S., Mubin M.H.A., Chaudhary K., Jalil M.A., Ali J., Yupapin P. (2014). Detection ofSalmonella bacteriumin drinking water using microring resonator. Artif. Cells Nanomed. Biotechnol..

[21] Baaske M.D., Foreman M.R., Vollmer F. (2014). Single-molecule nucleic acid interactions monitored on a label-free microcavity biosensor platform. Nat. Nanotechnol..

[22] Squires T.M., Messinger R.J., Manalis S.R. (2008). Making it stick: Convection, reaction and diffusion in surface-based biosensors. Nat. Biotechnol..

[23] Marshall B.T., Long M., Piper J.W., Yago T., McEver R.P., Zhu C. (2003). Direct observation of catch bonds involving cell-adhesion molecules. Nat. Cell Biol..

[24] Gou Y., Jia Y., Wang P., Sun C. (2018). Progress of Inertial Microfluidics in Principle and Application. Sensors.

[25] Van Oijen A.M., Blainey P.C., Crampton D.J., Richardson C.C., Ellenberger T., Xie X.S. (2003). Single-Molecule Kinetics of Exonuclease Reveal Base Dependence and Dynamic Disorder. Science.

[26] Moffitt J.R., Chemla Y.R., Smith S.B., Bustamante C. (2008). Recent Advances in Optical Tweezers. Annu. Rev. Biochem..

[27] Lionnet T., Allemand J.-F., Revyakin A., Strick T.R., Saleh O.A., Bensimon D., Croquette V. (2011). Single-Molecule Studies Using Magnetic Traps. Cold Spring Harb. Protoc..

[28] Nakano A., Ros A. (2013). Protein dielectrophoresis: Advances, challenges, and applications. Electrophoresis.

[29] Ding J., Lawrence R.M., Jones P.V., Hogue B.G., Hayes M.A. (2016). Concentration of Sindbis virus with optimized gradient insulator-based dielectrophoresis. Analyst.

[30] Herrmann N., Neubauer P., Birkholz M. (2019). Spiral microfluidic devices for cell separation and sorting in bioprocesses. Biomicrofluidics.

[31] Abt V., Gringel F., Han A., Neubauer P., Birkholz M. (2020). Separation, Characterization, and Handling of Microalgae by Dielectrophoresis. Microorganisms.

[32] Viefhues M., Eichhorn R. (2017). DNA dielectrophoresis: Theory and applications a review. Electrophoresis.

[33] Camacho-Alanis F., Ros A. (2015). Protein dielectrophoresis and the link to dielectric properties. Bioanalysis.

[34] Li M., Li W.H., Zhang J., Alici G., Wen W. (2014). A review of microfabrication techniques and dielectrophoretic microdevices for particle manipulation and separation. J. Phys. D Appl. Phys..

[35] Pethig R. (2016). Review—Where Is Dielectrophoresis (DEP) Going?. J. Electrochem. Soc..

[36] Washizu M., Suzuki S., Kurosawa O., Nishizaka T., Shinohara T. (1994). Molecular dielectrophoresis of biopolymers. IEEE Trans. Ind. Appl..

[37] Hölzel R., Calander N., Chiragwandi Z., Willander M., Bier F.F. (2005). Trapping Single Molecules by Dielectrophoresis. Phys. Rev. Lett..

[38] Otto S., Kaletta U., Bier F.F., Wenger C., Hölzel R. (2014). Dielectrophoretic immobilisation of antibodies on microelectrode arrays. Lab Chip.

[39] Laux E.-M., Bier F.F., Holzel R. (2018). Electrode-based AC electrokinetics of proteins: A mini-review. Bioelectrochemistry.

[40] Li S., Cui H., Yuan Q., Wu J., Wadhwa A., Eda S., Jiang H. (2014). AC electrokinetics-enhanced capacitive immunosensor for point-of-care serodiagnosis of infectious diseases. Biosens. Bioelectron..

[41] Freedman K.J., Otto L.M., Ivanov A.P., Barik A., Oh S.-H., Edel J.B. (2016). Nanopore sensing at ultra-low concentrations using single-molecule dielectrophoretic trapping. Nat. Commun..

[42] Jäger M., Becherer T., Bruns J., Haag R., Petermann K. (2016). Antifouling coatings on SOI microring resonators for bio sensing applications. Sensors Actuators B Chem..

[43] Duarte V.C., Prata J.G., Ribeiro C.F., Nogueira R.N., Winzer G., Zimmermann L., Walker R., Clements S., Filipowicz M., Napierała M. (2019). Modular coherent photonic-aided payload receiver for communications satellites. Nat. Commun..

[44] Duarte V.C., Ribeiro C.F., Prata J.G., Winzer G., Petousi D., Zimmermann L., Nogueira R., Drummond M.V. (2018). Reconfigurable monitoring and control system for tunable optical delay lines. Opt. Lett..

[45] Sharma A., Han C.-H., Jang J. (2016). Rapid electrical immunoassay of the cardiac biomarker troponin I through dielectrophoretic concentration using imbedded electrodes. Biosens. Bioelectron..

[46] Gong J.-R. (2010). Label-Free Attomolar Detection of Proteins Using Integrated Nanoelectronic and Electrokinetic Devices. Small.

[47] Jäger M., Bruns J., Ehrentreich-Förster E., Petermann K. (2013). Arrays of Individually Addressable SOI Micro Ring Resonators for Bio Sensing. Integr. Photonics Res. Silicon Nanophotonics Photonics Switch..

[48] Moock P., Kasper L., Jäger M., Stolarek D., Richter H., Bruns J., Petermann K. (2018). TDM-controlled ring resonator arrays for fast, fixed-wavelength optical biosensing. Opt. Express.

[49] Schwelb O. (2004). Transmission, Group Delay, and Dispersion in Single-Ring Optical Resonators and Add/Drop Filters—A Tutorial Overview. J. Light. Technol..

[50] Lima L.F., Vieira A.L., Mukai H., Andrade C.M., Fernandes P.R. (2017). Electric impedance of aqueous KCl and NaCl solutions: Salt concentration dependence on components of the equivalent electric circuit. J. Mol. Liq..

[51] Myers T.L., Tonkyn R.G., Danby T.O., Taubman M.S., Bernacki B.E., Birnbaum J.C., Sharpe S.W., Johnson T.J. (2017). Accurate Measurement of the Optical Constants n and k for a Series of 57 Inorganic and Organic Liquids for Optical Modeling and Detection. Appl. Spectrosc..

[52] Saunders J.E., Sanders C., Chen H., Loock H.-P. (2016). Refractive indices of common solvents and solutions at 1550 nm. Appl. Opt..

[53] Kittler M., Yu X., Vyvenko O., Birkholz M., Seifert W., Reiche M., Wilhelm T., Arguirov T., Wolff A., Fritzsche W. (2006). Self-organized pattern formation of biomolecules at silicon surfaces: Intended application of a dislocation network. Mater. Sci. Eng. C.

[54] Kim D., Sonker M., Ros A. (2018). Dielectrophoresis: From Molecular to Micrometer-Scale Analytes. Anal. Chem..

[55] Hölzel R., Pethig R. (2020). Protein Dielectrophoresis: I. Status of Experiments and an Empirical Theory. Micromachines.

[56] Wirth J.C. (2019). Engineering Sensitivity: An Optical Optimization of Ring Resonator Arrays for Label-Free Whole Bacterial Sensing. Ph.D. Thesis.

[57] Ramachandran A., Wang S., Clarke J., Ja S., Goad D., Wald L., Flood E., Knobbe E., Hryniewicz J., Chu S. (2008). A universal biosensing platform based on optical micro-ring resonators. Biosens. Bioelectron..

[58] Kirschner A.K.T. (2016). Determination of viable legionellae in engineered water systems: Do we find what we are looking for?. Water Res..

