# Supplementary material for: An Approach to Ring Resonator Biosensing Assisted by Dielectrophoresis: Design, Simulation and Fabrication
Source: Micromachines (Basel). 2020 Oct 22;11(11):954. doi: 10.3390/mi11110954 (PMC7690605; doi:10.3390/mi11110954)
Supplement: Supplementary file 1 [file micromachines-11-00954-s001.pdf]

# An approach to ring resonator biosensing assisted by dielectrophoresis: Design, simulation and fabrication

**Anders Henriksson, Laura Kasper, Matthias Jäger, Peter Neubauer and Mario Birkholz**

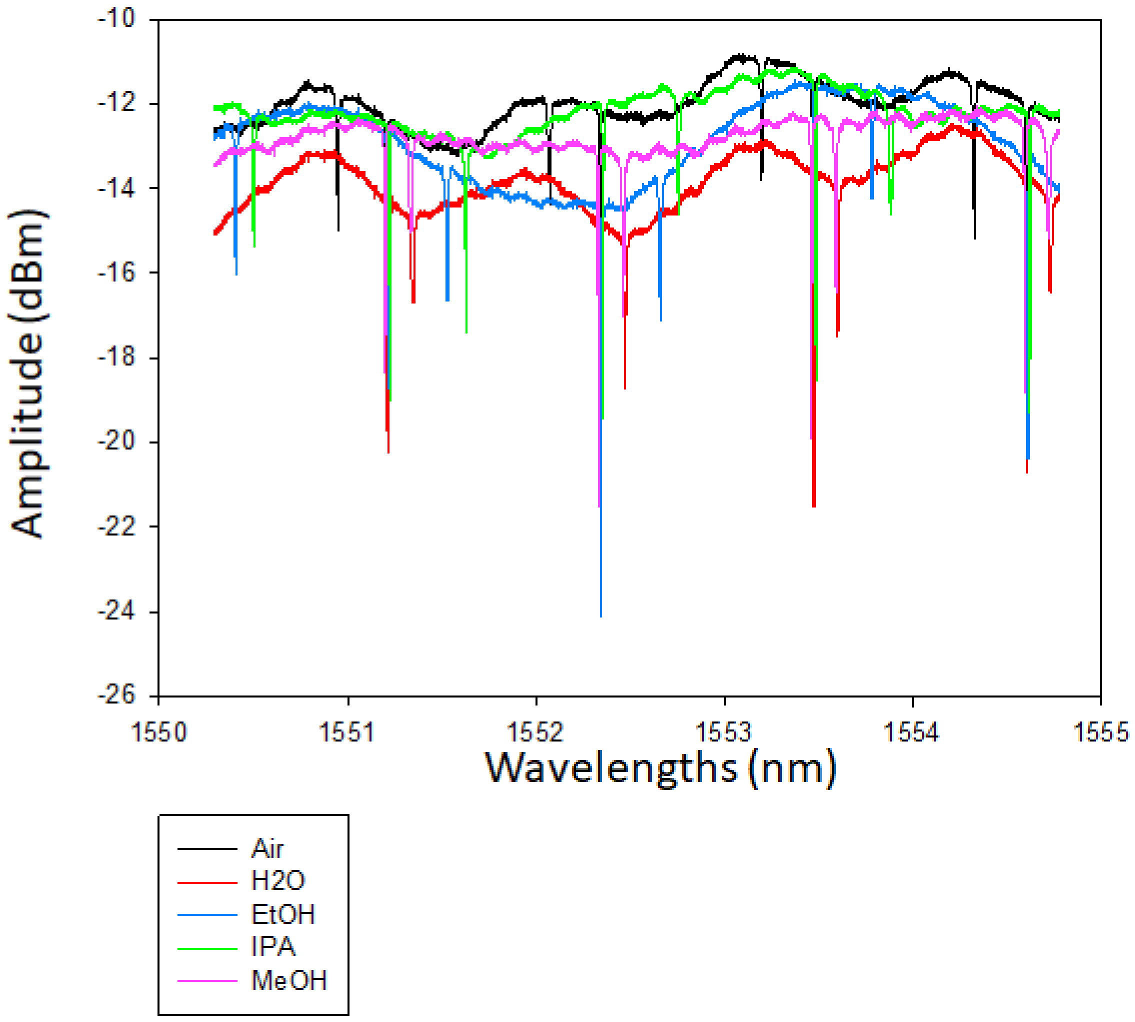


**Figure 1.** Typical transmission spectrum of ringresonators with coplanar electrode configuration.

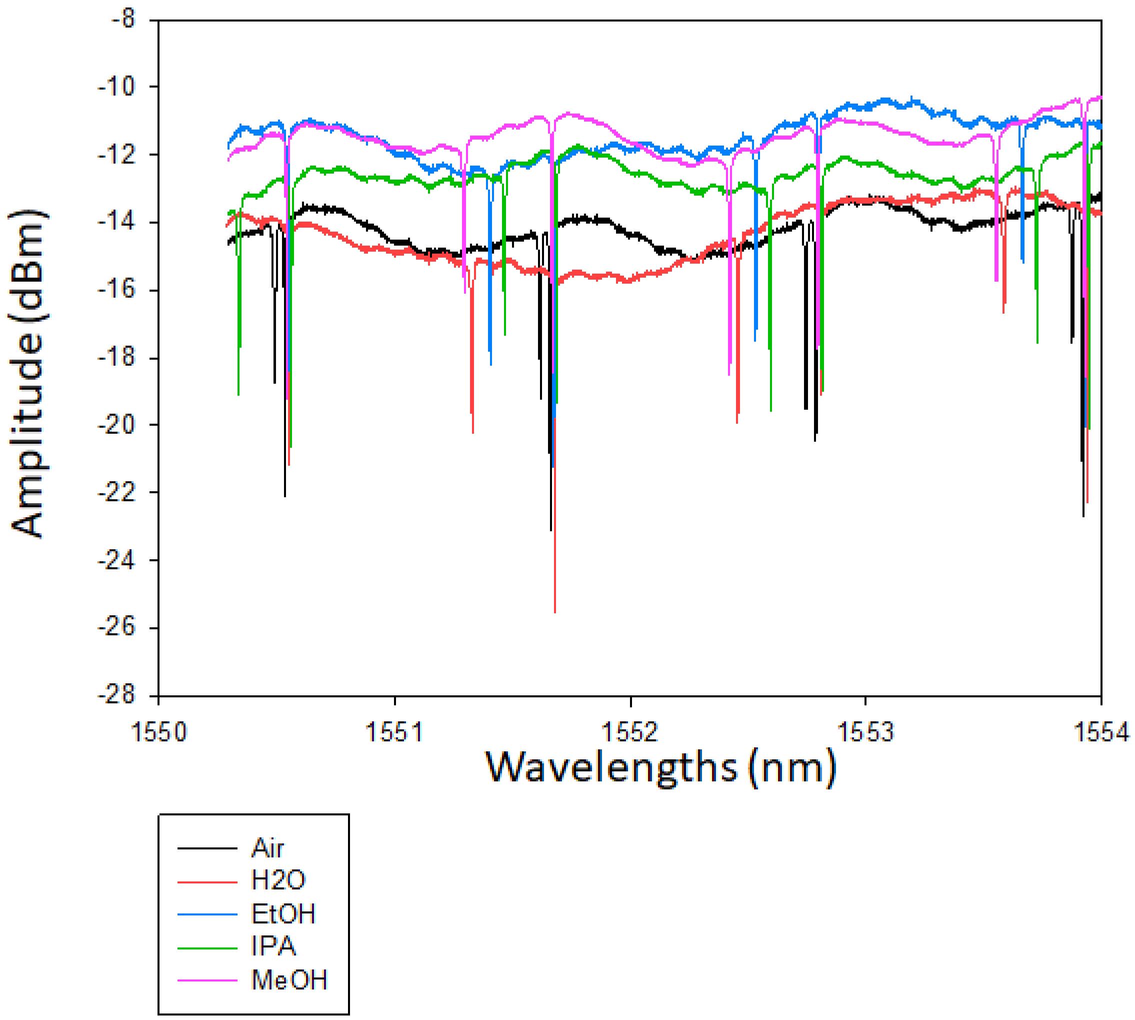


**Figure 2.** Typical transmission spectrum of ringresonator with top-bottom electrode configuration.

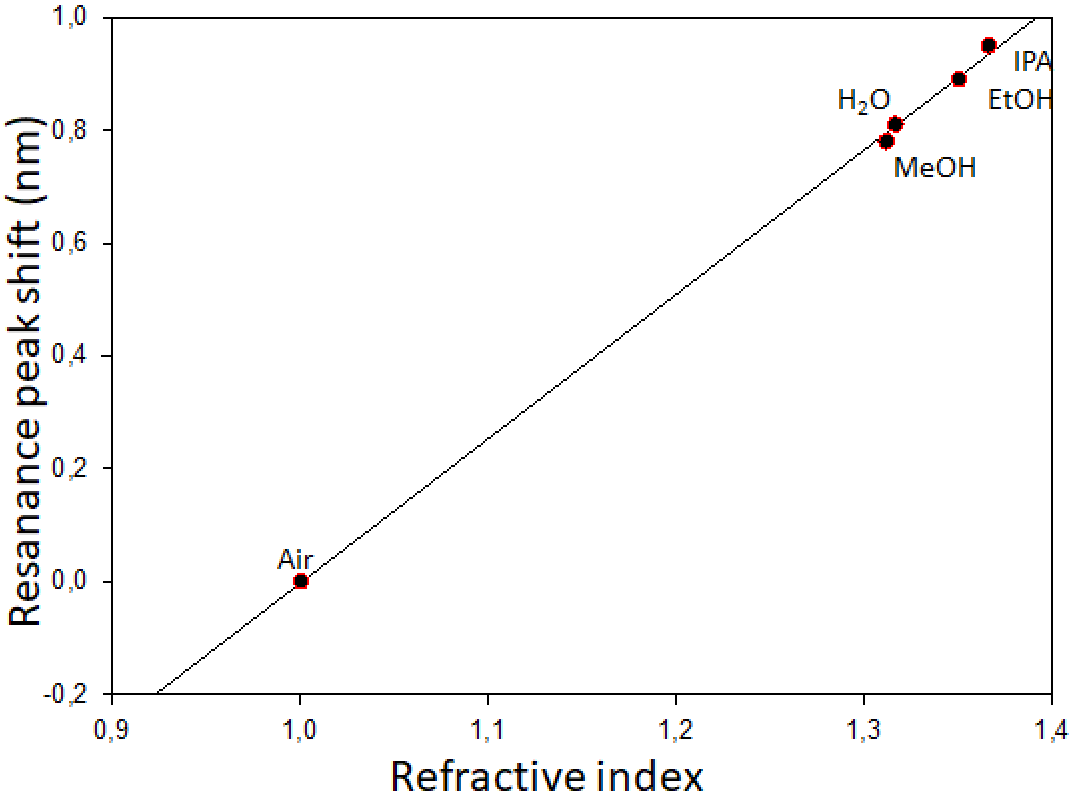


**Figure 3.** The resonance peak shift of the ring resonators with coplanar electrodes exposed to MeOH ( n = 1..3118), $H_2O$ (n = 1.3164), EtOH (n = 1.3503), and isopropanol (n = 1.3661) normalized to the peak position in air.
